# Effectiveness of nonpharmacological interventions for reducing postpartum fatigue: a meta-analysis

**DOI:** 10.1186/s12884-021-04096-7

**Published:** 2021-09-14

**Authors:** Jialu Qian, Shiwen Sun, Lu Liu, Xiaoyan Yu

**Affiliations:** 1 Zhejiang University School of Medicine, Zhejiang, Hangzhou China; 2 Department of Obstetrics and Gynecology, Women’s Hospital School of Medicine, Zhejiang University, 1st Xueshi Road, Hangzhou, 310006 Zhejiang Province China

**Keywords:** Fatigue, Postpartum, Depression, Nonpharmacological interventions, Meta-analysis

## Abstract

**Background:**

Postpartum fatigue is the most common issue among postnatal women and it could not only seriously affect the health of mothers but also bring about adverse impacts on their offspring. This meta-analysis aims to synthesize nonpharmacological evidence and evaluate the effectiveness of interventions for reducing postpartum fatigue among puerperae.

**Methods:**

The Cochrane Library, PubMed, Embase, Web of Science, PsycINFO, CINAHL and ProQuest databases were searched for papers published from inception until June 2021. Grey literature was searched using OpenGrey. Randomized controlled trials (RCTs) or controlled clinical trials (CCTs) evaluating nonpharmacological interventions conducted during 0 ~ 78 weeks postpartum for fatigue reduction were eligible for inclusion. The methodological quality of the included studies was independently assessed by two reviewers using the Cochrane risk-of-bias tool and the risk of bias in nonrandomized studies of interventions. Cohen’s kappa coefficient was used to measure inter-rater agreement. The meta-analysis was conducted using Review Manager 5.3.

**Results:**

Seventeen published clinical trials matched the eligibility criteria and ten studies involving 1194 participants were included in this meta-analysis. The intervention start time varied from immediately postpartum care to 1 year after delivery, and duration ranged from 1 day to 3 months. The results revealed that exercise (SMD = − 1.74, 95% CI = -2.61 to − 0.88) and drinking tea (MD = − 3.12, 95% CI = -5.44 to − 0.80) resulted in significant improvements in women’s postpartum fatigue at postintervention. Drinking tea may have beneficial effects on depression (MD = − 2.89, 95% CI = -4.30 to − 1.49). Positive effects of psychoeducational interventions on postpartum fatigue or depression were not observed. Physical therapies including mother-infant skin-to-skin contact, taking warm showers and breathing lavender oil aroma were used for reducing postpartum fatigue. No significant risk of publication bias was found. Small number of included studies and sample sizes, not time-matched conditions of control groups, high heterogeneity and the risk of bias within the included studies were the main limitations of our review.

**Conclusions:**

This review provides evidence that exercise and drinking tea may be effective nonpharmacological interventions for relieving postpartum fatigue. More effective and targeted exercise programs need to be further studied. Rigorous RCTs of drinking tea are needed. Caution is required when interpreting the findings due to the limitations of our study. Further studies are still needed to validate our findings and increase confidence in the results.

**Supplementary Information:**

The online version contains supplementary material available at 10.1186/s12884-021-04096-7.

## Background

Postpartum fatigue is considered the most common issue that postnatal women confront when they transition to motherhood [1]. Postpartum fatigue is described as overwhelming feelings of exhaustion, and decreases in physical and mental capacity [2]. It is highest in the days after giving birth. These symptoms may disturb approximately 64% of mothers in their postpartum stage [2]. It was reported that 38.8, 27.1 and 11.4% of women perceived fatigue at 10 days, 1 month and 3 months after delivery, respectively, which indicates that the influence of postpartum fatigue on puerperae is general and persistent. Negative psychological symptoms (e.g., depression, anxiety and stress), sleep problems and less effective parenting behaviours are closely associated with the severity of postpartum fatigue [3–5]. Importantly, previous research demonstrated that postpartum fatigue could not only seriously affect the maternal health of mothers but also bring about adverse impacts on their offspring [6]. Experiences of fatigue could negatively affect breastmilk production, maternal-infant attachment and interactions [5, 7, 8], thereby delaying the development of babies [5].

Hence, it is significant to deliberately avert and relieve fatigue during the postpartum period via effective approaches. In fact, interventions for reducing postpartum fatigue have particular advantages. They are not only important to puerperae’ physical relief but also have potential benefits on the improvement of maternal mental health. Dennis et al. [9] suggested that preventing postpartum depression could start from the perspective of fatigue management. Compared with recommended psychotherapy, fatigue management is less stigmatising and may be an acceptable first step for women to seek assistance and receive treatment for psychological issues [9].

Postpartum fatigue is an ongoing research issue. However, it is minimally examined and there is little evidence for the effective treatment of postpartum fatigue [3, 9]. On the positive side, there is rapidly growing interest in this area. Although studies of nonpharmacological interventions conducted in puerperae with the aim of reducing fatigue are accumulating, different interventions may lead to varied effects [10–13]. Previous relevant systematic reviews and meta-analyses have clarified the positive relationship of postpartum fatigue and depression [3], the predictive factors of postpartum fatigue [1] and the effects of exercise on pregnancy and postpartum fatigue [14].

To the best of our knowledge, there is currently no meta-analysis of nonpharmacological interventions specific for postpartum fatigue. Given that postpartum fatigue duration is in direct proportion to the severity of related health effects, it ranges from the first postpartum weeks to several months [15]. A previous systematic review [3] indicated that the longest follow-up time of postpartum fatigue is 78 weeks after delivery. Therefore, we defined postpartum as 0 to 78 weeks after giving birth in our study. The aim of this study was to identify existing nonpharmacological interventions offered to postnatal women and to examine their effectiveness for relieving postpartum fatigue.

## Methods

### Protocol and registration

The protocol has been registered in the International Prospective Register of Systematic Reviews (PROSPERO [RRID: SCR_019061]); registration number CRD42021234869.

### Study design

This meta-analysis has been reported following the Preferred Reporting Items for Systematic Reviews and Meta-Analysis statement (PRISMA [RRID: SCR_018721]). The PRISMA checklist can be seen in Additional file 1.

### Search strategy

The focus of the review was to examine nonpharmacological interventions for fatigue reduction in postpartum women. Seven electronic databases, including the Cochrane Library, PubMed, Embase, Web of Science, PsycINFO, CINAHL and ProQuest, were searched for articles published from inception until June 2021 without time restrictions. An additional search of OpenGrey was conducted for grey literature. First, we identified main concepts including “postpartum”, “fatigue” and “clinical trial” using the PICOS (Population, Intervention, Comparison, Outcome and Study design) search tool. Then for each concept, a combination of Medical Subject Heading, Emtree terms and free terms [e.g., (postnatal OR postpartum OR delivery OR childbirth OR birth OR parturition OR labour OR pregnancy) AND (fatigue OR mental fatigue OR lassitude OR exhaust* OR tiredness OR tired*) AND (clinical trial OR randomized controlled trial OR controlled clinical trial OR cohort)] was used. The final search strategies applied are shown in Additional file 2. Moreover, a manual search of reference lists was performed to thoroughly identify relevant studies that were missed. Two authors (JLQ and SWS) performed the search process independently.

### Eligibility/exclusion criteria for selecting studies

The inclusion criteria of this review were identified based on PICOS: (1) Participants: women aged 18 years or over who had a healthy pregnancy without postnatal complications; (2) Intervention: nonpharmacological interventions conducted during 0 ~ 78 weeks postpartum with the primary or secondary aim of decreasing fatigue symptoms. The intervention setting, frequency, timing and duration were not limited; (3) Comparison: usual care, placebo, waitlist or no interventions; (4) Outcomes: the primary efficacy outcome was postpartum fatigue estimated as the rate or mean severity of fatigue; The second efficacy outcome was psychological variables, such as depression, anxiety or stress; (5) Study design: Clinical trials adopting randomized controlled trials (RCTs), quasi-experimental, before-and-after or prospective cohort study designs; and (6) Original articles published in English. The exclusion criteria were as follows: (1) duplicated publications (only the one with the most participants was included); and (2) studies without sufficient data to be extracted.

After removing duplicates, two authors (JLQ and SWS) independently screened the studies according to the inclusion criteria in 2 steps: 1) title and abstract screening and 2) full-text screening. A third author (LL) was consulted to reach a consensus when there was any uncertainty about the inclusion of an article.

### Data extraction

A standardized data extraction sheet was used to extract important information from the included studies. The extracted data included first author, publication year, country, study design, population, sample size (trial/control), intervention details (e.g., type, start time, duration and frequency), control, evaluation time points, assessment tools and outcomes. Two authors (JLQ and SWS) independently extracted the data, and any inconsistencies were resolved by a third author (LL).

### Risk of bias assessment

For RCTs, the Cochrane risk-of-bias tool was used for the quality assessment [16]. Studies were assessed based on seven criteria (random sequence generation, allocation concealment, blinding of participants and researchers, blinding of outcome assessor, incomplete outcomes data, selective reporting and other bias). Bias was evaluated as a judgement (high or low or unclear), and then each included study was rated as having a high, moderate or low risk of bias. The Risk of Bias in Nonrandomized Studies of Interventions was used to assess the risk of bias of non-RCTs [17]. Seven domains are evaluated in this tool (confounding, selection of participants into the study, classification of intervention, deviation from the intended interventions, missing data, measurements of outcomes and selection of the reported results). An inter-rater reliability score using cohen’s kappa [18] for risk of bias was used. Cohen’s kappa coefficient is a statistical measure of inter-rater agreement (< 0: poor agreement; 0.0–0.20: slight agreement; 0.21–0.40: fair agreement; 0.41–0.60: moderate agreement; 0.61–0.80: substantial agreement; 0.81–1.0: almost perfect agreement). Two authors (JLQ and SWS) assessed the risk of bias and evidence quality separately.

### Data analysis

We performed a meta-analysis utilizing Review Manager 5.3 (RRID: SCR_003581). For continuous outcomes, we calculated mean differences (MDs) and 95% confidence intervals (CIs) if the outcomes were measured using the same tool. We used standardized mean differences (SMDs) and 95% CIs to combine studies when the same outcome was measured by adopting different tools [19]. For the SMD, ≤0.20, =0.50, and ≥ 0.80 are designated as small, moderate and large effect sizes, respectively. The heterogeneity among the analysed trials was examined by standard chi-square and I-square statistics. If the *P* value was > 0.1 or I^2^ < 50%, it indicated that there was no observed heterogeneity, and the researchers employed a fixed-effects model to combine the study results. If not, a random-effects model analysis was used [20]. Publication bias was examined using Egger’s test because it is not appropriate to use funnel plots when analysing fewer than 10 studies [21].

## Results

**Fig. 1:**
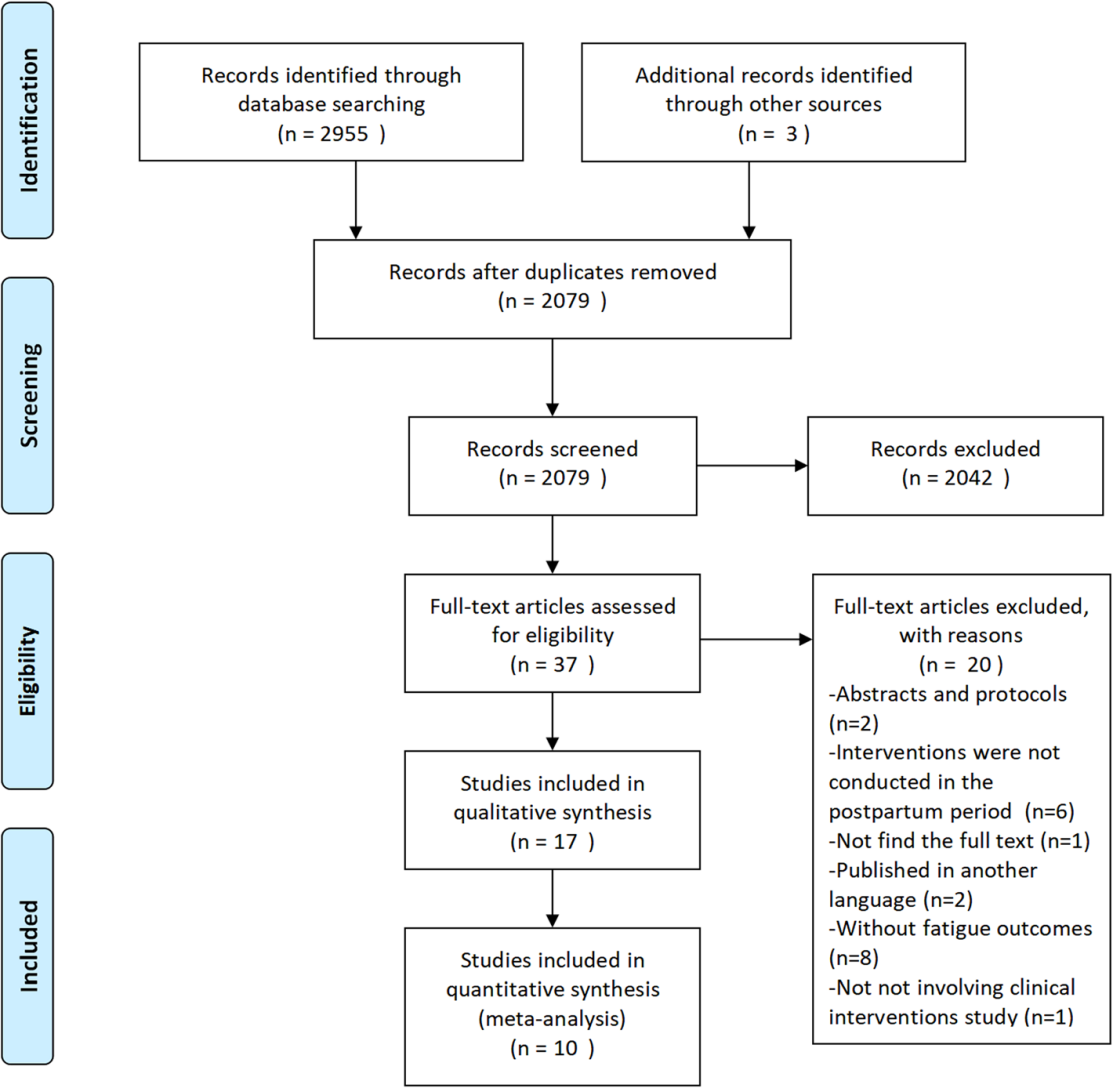
PRISMA flow diagram

The identified citations were imported into EndNote software (RRID:SCR_014001) and screened for duplicates. The initial search of 7 databases revealed 2981 references. A search of reference lists and OpenGrey revealed 3 other relevant studies. After removing 879 duplicates, the titles and abstracts of the remaining 2105 articles were screened, which excluded 2068 articles and left 37 full-text articles that were reviewed for eligibility. Twenty studies were excluded for being abstracts and protocols (*n* = 2), interventions were not conducted in the postpartum period (*n* = 6), could not find the full text (*n* = 1), studies not published in English (n = 2), studies without fatigue outcomes (*n* = 8) and studies not involving clinical interventions (n = 1). Ultimately, 17 studies were included in this review, and 10 studies met the criteria for meta-analysis. A PRISMA flow diagram illustrating the detailed study selection process is shown in Fig. 1.

### Study characteristics

The study characteristics are summarized in Table 1. Studies were published between 2003 ~ 2020. The 17 included articles were conducted in different countries: China (*n* = 5), Iran (*n* = 4), the USA (*n* = 2), Canada (n = 2), Turkey (n = 2), Australia (*n* = 1) and the UK (n = 1). A total of 2142 participants were included in this review. The sample size ranged from 27 to 356 participants in each trial. Eleven of the 17 included studies employed a randomized controlled trial design.

**Table 1:** Study characteristics (*n* = 17)

First author (publication year)	Country	Study design	Population	Sample size (intervention/control)	Intervention	Frequency	Intervention start time and duration	Control	Evaluation time points	Fatigue assessment tools	Psychological assessment tools
**Exercise (n = 5)**											
Ashrafinia [22] (2015)	Iran	RCT	Primiparous women, EPDS<10	80 (40/40)	Pilates home exercises	5 times a week (30 min/session)	Intervention was started 72 h after delivery and continued for 8 weeks	Routine care	Baseline, 4 weeks, 8 weeks postpartum	MFI-20	/
Dritsa [23] (2008)	Canada	RCT	Women in the postpartum (4–38 weeks), EPDS≧10	88 (46/42)	Aerobic exercise, stretching and strength exercises	60–120 min/week, 4 times	Intervention was started 4–38 weeks (mean: 10.96) postpartum and continued for 12 weeks	Assessing exercise participation once a month	Baseline, post-treatment and 3 months post-treatment	MFI-20	/
Ko [24] (2008)	China	A control group pre−/post-program design	Postpartum women who were “doing-the-month”	61 (31/30)	A low-intensity exercise program	6 sessions	Intervention was started from the first week postpartum and continued for 3 weeks	Usual care	Before and after the program	FSC	CES-D Scale
Lee [25] (2016)	UK	RCT	Postnatal women who had given birth between 6 weeks and one year previously	65 (33/32)	Physical activity consultations and a moderate-intensity walking programme	30–55 min/time, one session each week	Intervention was started at 6 weeks to one year after delivery and continued for 10 weeks	Received a leaflet with information on physical activity	Baseline, three-month and six-month follow-up.	VAS-F	AGWBI
Yang [12] (2019)	China	RCT	Postnatal women who had vaginal delivery, EPDS ≦ 10	140 (70/70)	Moderate-intensity aerobic exercise	At least 3 times a week (15 min per section)	Intervention was started at 6 weeks after childbirth and continued for 12 weeks	Usual care	Baseline, at 4 weeks and 12 weeks postintervention	PFS	PSS, EPDS
**Psychoeducational intervention (n = 7)**											
Doering [15] (2018)	USA	Quasi-experimental	Postpartum women who had a healthy singleton newborn	27 (15/12)	Helping U Get Sleep. Self-management intervention was offered via home visit and phone calls	A home visit and 4 phone calls	Intervention was started at the third week postpartum and continued for 3 weeks.	Health education related to sleep	Baseline, postpartum weeks 4, 6 and 9	MFSC	/
Gholami [26] (2017)	Iran	A control group pre−/post-program design	Women who had given birth	120 (40/40/40)	Educational behavioural interventions including instructions of the health approaches, relaxation techniques (face-to-face/ e-learning)	3 times	Intervention was started on 10 days after delivery and continued for 50 days	Usual care	Before and after the intervention	FSS	/
Giallo [27] (2014)	Australia	RCT	Mothers who had a child younger than the age of 6 months	202 (63/67/72)	Intervention group 1 (professionally led support): offer workbook, home visit and phone calls; Intervention group 2 (self-directed written): read workbook containing information about fatigue	A workbook, home visit, and 3 telephone support calls; reading the workbook	Intervention was started within 6 months after delivery and continued for 4 weeks	Usual care	Baseline, 6 and 12 weeks after the baseline	FAS, FSS,	DASS-21
Milani [28] (2017)	Iran	Clinical trial	Healthy postpartum mothers, EDPS< 10	276 (92/184)	A comprehensive postpartum home care program giving instructions of personal hygiene, mental, psychological, and sexual health, oral and dental health and so on	2 home visits	Intervention was started immediately postpartum care and continued for 60 days	Usual care	Pretest and posttest	Fatigue rate	EPDS
Ozcan [13] (2020)	Turkey	RCT	Primiparous women	117 (58/59)	Levin’s conservation model containing instructions of nutrition, sleep, breastfeeding and so on	8 sessions, each session lasted 60–120 min.	Intervention was started between postpartum 4th and 7th days and continued for 12 weeks	Usual care	Pretest and posttest	VAS-F	/
Stremler [29] (2013)	Canada	RCT	Primiparous women	246 (123/123)	Behavioural-educational sleep intervention containing sleep promotion strategies, sleep hygiene and so on	A 45–60 min meeting, a 20 page booklet, and 3 phone contacts.	Intervention was started before mothers’ discharge from hospital and continued for 4 weeks	Usual care	Baseline, 6 and 12 weeks	VAS-F	EPDS
Troy [30] (2003)	USA	Quasi-experimental	Healthy primiparous postpartum mothers	68 (32/36)	The Tiredness Management Guide containing a list of several techniques for postpartum fatigue management	Women in the intervention group were asked to use the TMG whenever they felt tired	Intervention was started at 2 weeks after delivery and continued for 4 weeks	Usual care	Fatigue was assessed six times per week, before going to bed and again on rising from Tuesday evening to Friday morning	VAS-F	/
**Drinking tea (n = 2)**											
Chang [31] (2015)	China	RCT	Postnatal women with poor sleep quality (PSQS score ≧16)	80 (40/40)	Drink one cup of chamomile tea	Every day	Intervention was started at 6 weeks after childbirth and continued for 2 weeks	Usual care	Baseline and at 2 and 4 weeks post intervention	PFS	EPDS
Chen [32] (2015)	China	RCT	Postnatal women with poor sleep quality (PSQS score ≧16)	80 (40/40)	Drink one cup of Lavender tea	Every day	Intervention was started at 6 weeks after childbirth and continued for 2 weeks	Usual care	Baseline, 2-week posttest and 4-week posttest	PFS	EPDS
**Physical therapy (n = 3)**											
Funda [10] (2020)	Turkey	RCT	Primiparous and had a vaginal delivery at the 37th to 40th weeks of gestation	80 (40/40)	Mother-infant skin-to-skin contact	1 time	Intervention was started from the first hour following the delivery and continued for 30 min	Usual care	Before and after the intervention	VAS-F	/
Hsieh [33] (2017)	China	Quasi-experimental	Healthy postpartum women	356 (94/264)	Take warm showers	1 time	Intervention was started on the second postpartum day and continued for 20 min	Usual care	The first postpartum day and the second postpartum day	PFS	/
Vaziri [11] (2017)	Iran	RCT	Primiparous women with normal vaginal delivery	56 (29/27)	Breathe lavender oil aroma	3 times, 10–15 min each time	Intervention was started immediately postpartum care and completed in 1 day	Sesame oil used as placebo	Baseline, after the first intervention and the tomorrow morning assessment	VAS-F	VAS for distress, PANAS

*Notes*: *RCT* Randomized clinical trial, *MFI-20* Multidimensional Fatigue Inventory, *PFS* Postpartum Fatigue Scale, *VAS-F* Visual analogue scale for fatigue, *FAS* Fatigue Assessment Scale, *MFSC* Modified Fatigue Symptoms Checklist, *FSC* Fatigue Symptom Checklist, *EDPS* Edinburgh Postnatal Depression Scale, *DASS-21* Depression, Anxiety and Stress Scale-21, *CES-D Scale* Chinese version of the Center for Epidemiologic Studies Depression Scale, *PANAS* Positive and Negative Affect Schedule, *PSS* Perceived Stress Scale, *AGWBI* Adapted General Well-Being Index

Postpartum interventions were grouped into four broad categories: exercise [12, 22–25] (*n* = 5), psychoeducational intervention [13, 15, 26–30] (*n* = 7), drinking tea [31, 32] (*n* = 2) and physical therapy [10, 11, 33] (*n* = 3). The intervention duration ranged from 1 day to 3 months. The intervention start time varied from immediately postpartum care to 1 year after delivery. The corresponding finish time of intervention ranged from parturition day to 1 year and 3 months postpartum. All included studies described the baseline assessments, with the scores for the intervention and control groups comparable at baseline. Additionally, the studies reported assessment scores immediately after the intervention and follow-up assessment scores at 1 month postintervention [31, 32], 6 weeks [15, 27, 29] and 9 weeks postintervention [15], 2 months postintervention [22], 3 months postintervention [23, 25, 27, 29] and 6 months [25] postintervention. These trials adopted the MFI-20, MFSC, PFS, VAS-F, FAS and FSC to evaluate the level of postpartum fatigue; the EDPS, DASS-21, CES-D, VAS for depression; and the PANAS and PSS were used to assess psychological variables such as anxiety and stress.

### Risk of bias in included studies

**Fig. 2:**
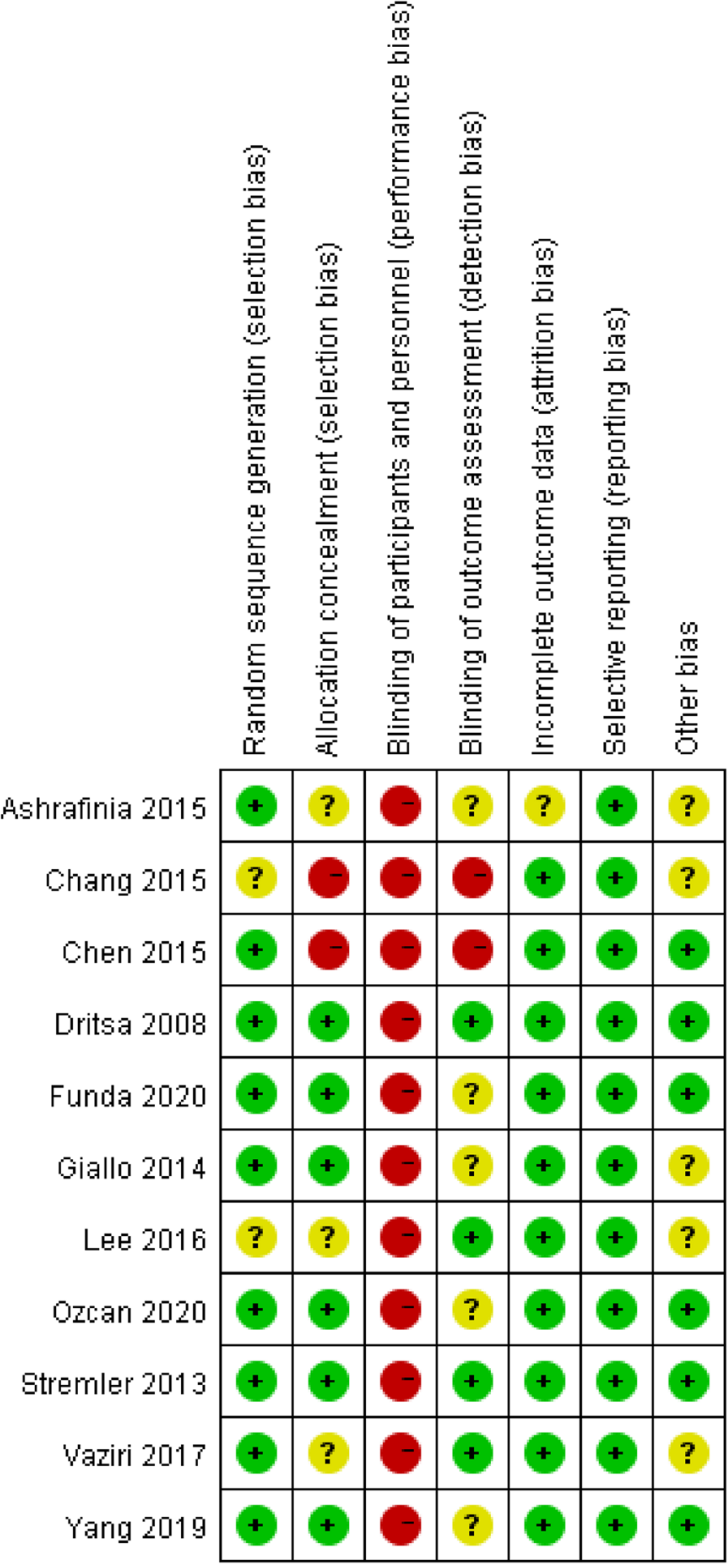
Risk of bias for individual RCTs

**Fig. 3:**
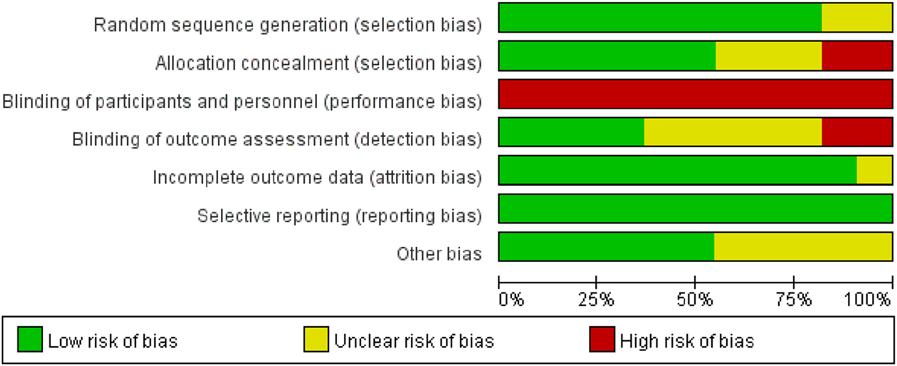
Risk of bias summaries for the included RCTs

The quality of the study designs was low to moderate overall. Several methodological limitations were observed in the critical appraisal. Quality assessments of the 11 included studies using an RCT design, with a risk of bias graph and risk of bias summary, are described in Fig. 2 and Fig. 3. All of the studies reported using randomization; however, two articles did not provide detailed information about the randomization method. Six studies reported sufficient details about allocation concealment. Blinding of the participants and researchers who delivered the interventions was not feasible because the interventions were easy to identify. Therefore, all of the studies are at a high risk of performance bias. Regarding detection bias, only four studies provided sufficient explanations. Except for one study, all RCT studies gave clear information about the incomplete outcome data. No reporting bias was found in the included RCTs. The results of the quality appraisal of the six nonrandomized studies are displayed in Table 2. Bias due to confounding factors, selection of participants into the study and classification of interventions were low for all included nonrandomized studies. Three articles were at moderate risk of bias due to deviations from the intended intervention. All of the studies were reasonably reported and addressed missing data. In regard to bias in the measurement of outcomes, considering that the interventions were not blinded to the participants, all studies were judged as at moderate risk of bias. Bias in selection of the reported results was not observed. According to the assessment results, the calculated cohen’s kappa was 0.74, which suggested that there was a substantial agreement between two raters.

**Table 2:** Results of the quality appraisal of nonrandomized studies (*n* = 6)

	Domain 1	Domain 2	Domain 3	Domain 4	Domain 5	Domain 6	Domain 7	Judgement
Doering (2018) [16]	Low	Low	Low	Low	Low	Moderate	Low	Moderate
Gholami (2017) [29]	Low	Low	Low	Low	Low	Moderate	Low	Moderate
Hsieh (2017) [34]	Low	Low	Low	Low	Low	Moderate	Low	Moderate
Ko (2008) [25]	Low	Low	Low	Moderate	Low	Moderate	Low	Moderate
Milani (2017) [30]	Low	Low	Low	Moderate	Low	Moderate	Low	Moderate
Troy (2003) [32]	Low	Low	Low	Moderate	Low	Moderate	Low	Moderate

*Notes*: Domain 1: Confounding; Domain 2: Selection of participants into the study; Domain 3: Classification of interventions; Domain 4: Deviations from intended interventions; Domain 5: Missing data; Domain 6: Measurement of outcomes; Domain 7: Selection of the reported results

### Meta-analysis results

We did not conduct a meta-analysis on the physical therapy interventions. Because there are only three studies and the types of intervention are completely different in each study. For the purposes of meta-analysis, three types of interventions were undertaken: exercise, psychoeducational interventions and drinking tea. Only one trial [28] reported the postpartum fatigue rate. Three trials [15, 23, 25] without sufficient original data for meta-analysis were thus not included. Ultimately, ten studies that reported the mean fatigue scores and standard deviations were included in the meta-analysis. For the secondary outcomes, since more than two studies reported depression, a statistical combination was performed regarding this psychological outcome.

### Effectiveness of exercise

Three studies examined the effects of exercise on fatigue among postnatal women [12, 22, 24]. Pilates exercise, aerobic exercise, yoga were chosen in this kind of intervention. The intervention start time varied from 72 h after delivery to 6 weeks postpartum, and the intervention duration ranged from 3 to 12 weeks.

**Fig. 4:**
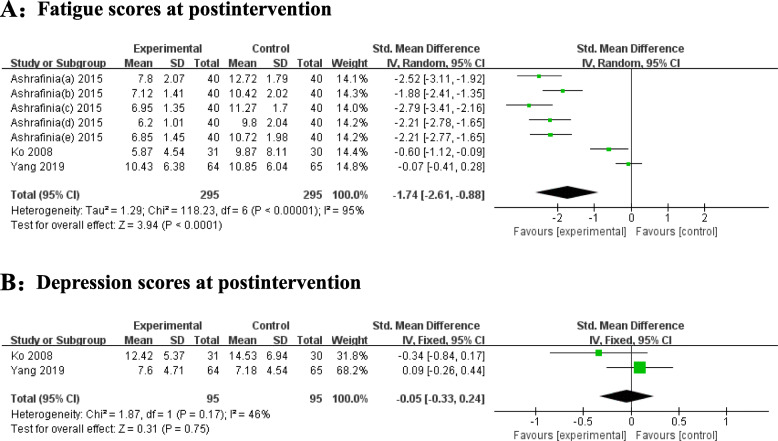
Forest plots for the effect of exercise on fatigue (A) and depression (B) postintervention. Notes: Ashrafinia et al.’s study assessed general fatigue (a), physical fatigue (b), reduced activity (c), reduced motivation (d), and mental fatigue (e)

Figure 4 shows the effect sizes of the exercise intervention in terms of fatigue and depression. For the primary outcome, three trials showed a postassessment of fatigue. The heterogeneity was I^2^ = 95% (*P* < 0.0001), so a random-effects model was applied. The pooled SMD was − 1.74 (95% CI = -2.61 to − 0.88, Z = 3.94, *P* < 0.0001), demonstrating that the exercise intervention had a better effect on decreasing fatigue symptoms. For the secondary outcome, depression data were presented in two trials. No significant effects on reducing depression were indicated. Significant heterogeneity was not detected (I^2^ = 46%, *P* = 0.17), so a fixed-effect model was used. The pooled SMD was − 0.05 (95% CI = -0.33 to 0.24, Z = 0.31, *P* = 0.75).

### Effectiveness of psychoeducational intervention

Five studies used psychoeducational intervention [13, 26, 27, 29, 30]. Psychoeducational intervention was most frequently conducted via home visits, leaflet/booklet and home calls in order to give women instructions of relaxation, sleeping exercises, nutrition and so on. The intervention start time varied from immediately postpartum care to 6 month postpartum, and duration ranged from 3 to 12 weeks.

**Fig. 5:**
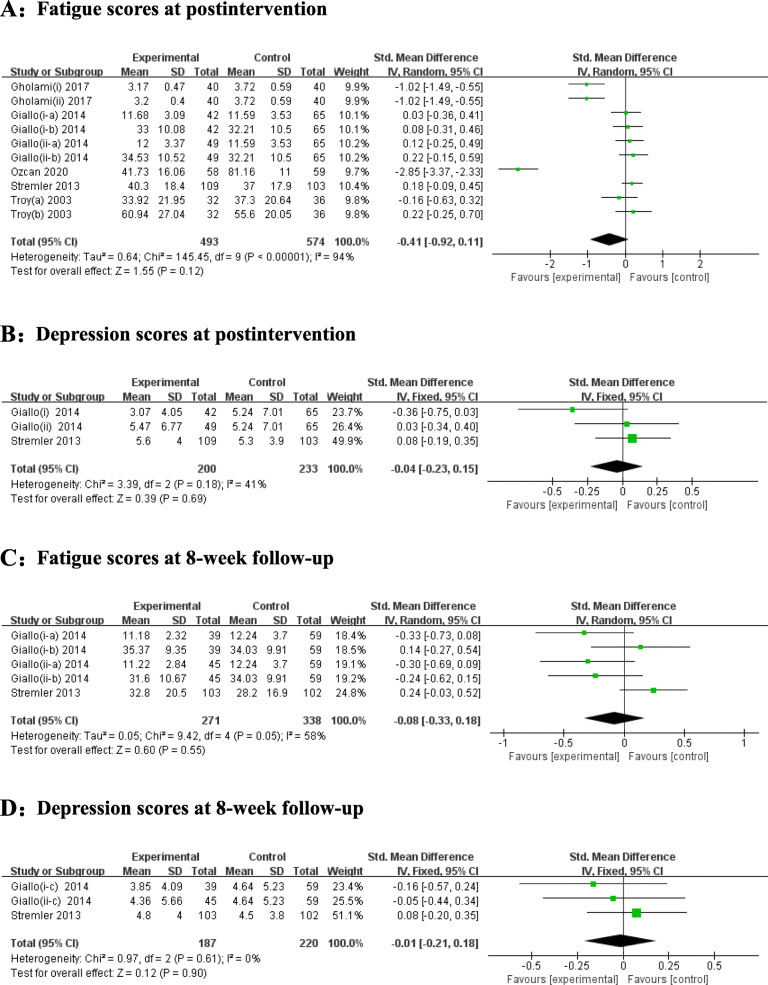
Forest plots for the effect of psychoeducational interventions on fatigue at postintervention (A), depression at postintervention (B), fatigue scores at the 8-week follow-up (C), and depression scores at the 8-week follow-up (D). Notes: Gholami et al.’s study included two intervention groups (i: e-learning and ii: face to face), which had assessment scores. Giallo et al.’s study included two intervention groups (i: professionally led telephone support and ii: self-directed written), and it used two independent scales (a: FAS and b: FSS) to assess fatigue symptoms in the same group. Troy et al.’s study examined morning fatigue (a) and evening fatigue (b)

Figure 5 displays the effect sizes of psychoeducational interventions according to fatigue and depression at postintervention and at the 8-week follow-up. Five studies displayed the results of psychoeducational interventions on fatigue. We found no significant difference between the two groups regarding fatigue. The heterogeneity was high (I^2^ = 94%, *P* < 0.0001), so a random-effects model was applied. The pooled SMD was − 0.41 (95% CI = -0.92 to 0.11, Z = 1.55, *P* = 0.12). Similarly, no significant effects on depression were noted in two trials presenting the depression data. We used a fixed-effects model because the heterogeneity was insignificant (I^2^ = 41%, *P* = 0.18). The pooled SMD was − 0.04 (95% CI = -0.23 to 0.15, Z = 0.39, *P* = 0.69).

Eight-week follow-up fatigue and depression assessments were presented in two trials. No significant effects on relieving fatigue and depression were indicated. For fatigue, we used a random-effects model because the heterogeneity was I^2^ = 58% (*P* = 0.05). The pooled SMD was − 0.08 (95% CI = -0.33 to 0.18, Z = 0.60, *P* = 0.55). In regard to 8-week follow-up depression scores, compared with the control groups, the participants in the intervention group did not experience significant changes in depression. There was no substantial evidence of high heterogeneity (I^2^ = 0%, *P* = 0.61); therefore, a fixed-effects model was used. The pooled SMD was − 0.01 (95% CI = -0.21 to 0.18, Z = 0.12, *P* = 0.90).

### Effectiveness of drinking tea

Two studies examined the effectiveness of drinking tea among participants who had poor sleep quality [31, 32]. Participants in the intervention group were required to drink one cup of chamomile tea every day for 2 weeks.

**Fig. 6:**
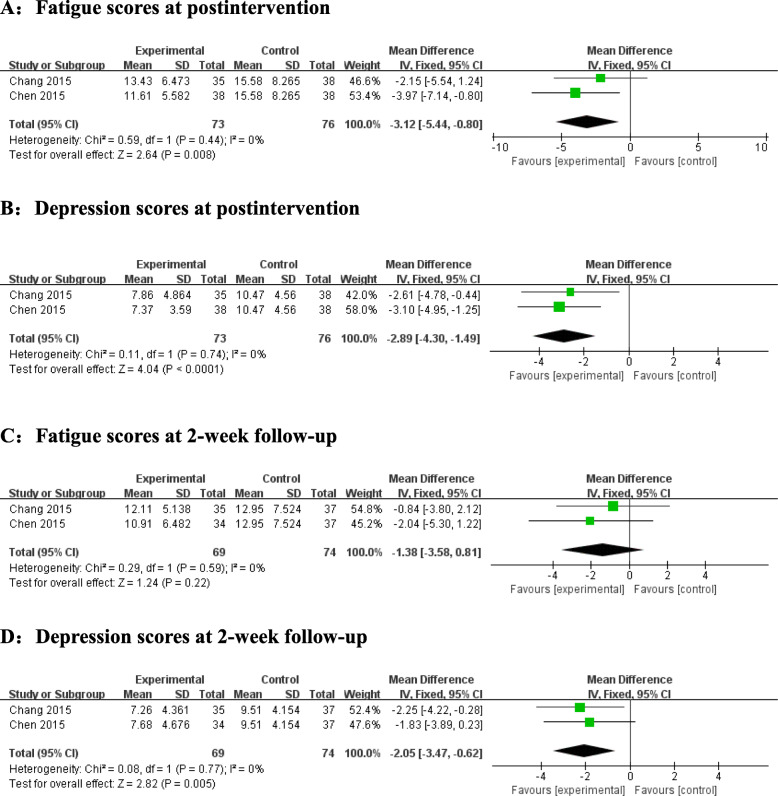
Forest plots for the effect of drinking tea on fatigue at postintervention (**A**), depression at postintervention (**B**), fatigue scores at the 2-week follow-up, (**C**) and depression scores at the 2-week follow-up (**D**)

The effectiveness of drinking tea on fatigue and depression at postintervention and at the 2-week follow-up were presented in two trials (Fig. 6). At postintervention, significant differences were noted between the intervention and control groups in regard to fatigue and depression. No significant heterogeneity was found in terms of fatigue and depression (I^2^ = 0%, *P* = 0.44; I^2^ = 0%, *P* = 0.74). Therefore, a fixed-effects model was used. The pooled MD was − 3.12 (95% CI = -5.44 to − 0.80, Z = 2.64, *P* = 0.008) and − 2.89 (95% CI = -4.30 to − 1.49, Z = 4.04, *P* < 0.0001).

At the 2-week follow-up, the results suggested that there were no significant differences between the intervention and control groups in regard to fatigue. There was no evidence of significant heterogeneity (I^2^ = 0%, *P* = 0.59). The pooled MD was − 1.38 (95% CI = -3.58 to 0.81, Z = 1.24, *P* = 0.22). The results showed that tea consumption had significant effects on depression at the 2-week follow-up. We used a fixed-effects model because the heterogeneity was I^2^ = 0% (*P* = 0.77). The pooled MD was − 2.05 (95% CI = -3.47 to − 0.62, Z = 2.82, *P* = 0.005).

### Descriptive summary on effectiveness of physical therapy

Three studies [10, 11, 33] involving 492 participants examined the effect of physical therapy interventions on postpartum fatigue. Mother-infant skin-to-skin contact, taking warm showers and breathing lavender oil aroma were approaches used as physical therapies. The intervention started within 1 to 2 days postpartum. The intervention frequency ranged from 1 to 3 times and each intervention time varied from 10 to 30 min. Funda et al. [10] investigated the efficacy of mother-infant skin-to-skin contact on postpartum fatigue. The infant was left in the prone position on the mother’s chest for 30 min in the first hour after the delivery. However, no significant differences between groups were found on postpartum fatigue. Hsieh et al. [33] instructed healthy postpartum women to take warm showers for approximately 20 min on the second day after delivery. The results indicated that warm showers significantly decreased postpartum fatigue in the intervention group compared to the control group. Vaziri et al. [11] tested breathing lavender oil aroma on managing postpartum fatigue among primiparous women. A significantly positive effect of this intervention on fatigue, distress, positive and negative moods was observed between groups.

### Publication bias

The potential publication bias was assessed by performing Egger’s linear regression test of exercise and psychoeducational interventions. For the primary outcome fatigue at postintervention, Egger’s test suggested no indication of publication bias of exercise (t = − 1.84, *p* = 0.317) and psychoeducational intervention (t = − 2.28, *p* = 0.052).

## Discussion

### Main findings

This review identified a total of seventeen published clinical trials examining nonpharmacological interventions (including exercise, psychoeducational interventions, drinking tea and physical therapy) on postpartum fatigue in postnatal women. Ten studies involving 1194 participants were included in the meta-analysis to examine the respective effectiveness of each type of intervention.

The findings of the meta-analysis suggest that exercise intervention significantly reduces symptoms of postpartum fatigue (SMD = − 1.74, 95% CI = -2.61 to − 0.88, Z = 3.94, *P* < 0.0001). This is consistent with the findings of previous studies [34, 35], which clarified the necessities and benefits of physical exercise for the overall health of postpartum women. A meta-analysis [36] indicated that postpartum exercise was beneficial for decreasing postpartum fatigue. However, postpartum women are still at high risk for physical inactivity and rarely understand how to engage in postpartum exercise [35]. The earliest start time of exercise intervention was 72 h after delivery among included studies and the exercise intervention usually continue for at least 3 weeks. The American College of Obstetricians and Gynaecologists (ACOG) suggested that physical activity, including performing muscle-toning exercises, could be restarted 6 weeks after childbirth if the delivery was uncomplicated [37]. Daily 20 ~ 30 min of regular moderate-to-intense exercise is recommended [38]. A previous study reported that supervised postpartum exercise lasting more than 8 weeks is suggested for reducing postpartum fatigue [14]. Therefore, clinical staff should give evidence-based instructions involving detailed start time, duration and frequency of postnatal exercises to help reduce women’s postpartum fatigue and facilitate their postpartum rehabilitation.

For depression, the results showed that there were no significant differences between the two groups (SMD = -0.05, 95% CI = -0.33 to 0.24, Z = 0.31, *P* = 0.75). This is inconsistent with the findings of a previous meta-analysis [39], which reported that exercise reduced symptoms of depression (SMD = -0.81, 95% CI = -1.53 to − 0.10) of mothers who had been diagnosed with depression. The insignificant results may be explained by the fact that the two studies [12, 24] in our review did not include participants with obvious depressed symptoms at baseline. Therefore, a significant reduction in depression was difficult to observe after the exercise intervention. It is worth noting that in Yang et al. [12]‘s study, women reported feelings of mood relaxation and pressure relief after the intervention, which suggested that exercise may have potential efficacy for women’s mental health. More research conducting exercise interventions among puerperae is needed to confirm the true effect of exercise on mothers’ psychological well-being.

Five studies performing psychoeducational interventions showed no obvious differences in postpartum fatigue and depression between the two groups at either the postintervention or 8-week follow-up. It’s worth noting that studies with different psychoeducational aims and strategies were combined in the meta-analysis, which may be the reason for significant heterogeneity. Psychoeducational interventions were offered to women immediately postpartum and were aimed at resolving various problems associated with postnatal health. This type of intervention was mostly delivered via home visits, leaflet/booklets and home calls. It is difficult to guarantee the good compliance of participants due to a lack of sufficient supervision and guidance. Therefore, it may be difficult to perform psychoeducational interventions effectively. With the development of rapid electronic technology, people’s requests for health services have also facilitated instant communication and promoted efficiency in the transmission of information [40]. A previous study reported that web-based interventions had better effects on improving postnatal depression than home-based postnatal psychoeducational interventions [41] and suggested that web-based interventions should be introduced to mothers for better postnatal care. Hence, psychoeducational interventions could be combined with internet technology [42] or smartphones [43] in the future to better manage the intervention implementation, observe the compliance of the participants and improve the intervention efficacy.

In terms of drinking tea, the results showed that there were significant differences in postpartum fatigue between the two groups at postintervention (MD = − 3.12, 95% CI = -5.44 to − 0.80, Z = 2.64, *P* = 0.008), but no significant differences were found at the 2-week follow-up. The positive effects of drinking tea on relieving depression were obvious at the postintervention (MD = − 2.89, 95% CI = -4.30 to − 1.49, Z = 4.04, *P* < 0.0001) and 2-week follow-up (MD = − 2.05, 95% CI = -3.74 to − 0.62, Z = 2.89, *P* = 0.005). Postnatal women were required to smell the aroma before drinking the tea. Aromatherapy has been used for pain and anxiety relief, relaxation, and creating a pleasant feeling in mothers [44–46], which could help to relieve fatigue and depressive emotions. Women in the intervention group reported the benefits of drinking chamomile tea to be facilitating emotional stability and relaxation and having an aromatic fragrance, which could calm restlessness, facilitate the postnatal paternity relationship, and alleviate postpartum fatigue [31]. However, the effects of drinking tea on fatigue was immediate but not long term in our findings. A possible reason for the short-lived effect is that the employed women had completed their maternity leave and returned to work at 2-week follow-up (10 weeks postpartum) [31, 32]. Therefore, additional measurement points with shorter intervals may be essential to confirm the actual duration of the therapeutic effects of drinking tea. In addition, how behaviour change wasn’t monitored in these two studies [31, 32]. It may lead to decreased treatment adherence, thereby influencing the long-term efficacy of the intervention. Future study should strengthen the supervision of intervention implementation so as to improve the efficacy. The effectiveness of this intervention should be considered cautiously due to lack of blinding in allocation concealment, intervention implementation and outcome assessment. High-quality RCTs further investigating the effect of drinking tea are needed.

Interventions of physical therapy were started within 1 to 2 days after childbirth under the guidance of medical staff and completed in 1 day. Physical therapies have the advantages of a short intervention time and good controllability. Warm showers, as a comfort measure, are closely associated with increased relaxation and tension reduction [47]. It is a safe and effective measure for healthy, labouring women who are experiencing physical and psychological issues [47]. The benefits of lavender oil on postpartum fatigue were reported in another RCT conducted among pregnant women [48]. Participants in either the lavender and footbath or lavender alone group showed that fatigue was improved significantly at 6 weeks postpartum. Although the efficacy of mother-infant skin-to-skin on fatigue was not observed in Funda et al.’s study, a recently published meta-analysis demonstrated that mother-infant skin-to-skin was a cost-effective, simple and feasible approach for postpartum depression [49]. Therefore, more studies should be conducted to understand the effectiveness of a certain physical therapy. Among the three included studies, only Vaziri et al.’s study examined the efficacy of lavender oil aroma for psychological outcomes, including distress and mood, and positive effects were observed. Considering that physical therapies are relatively safe, physical therapies such as footbaths [48], reflexology [50], warm showers [33] and lavender oil [11], which show potential effects on fatigue reduction and mental health improvement, could be used in combination to enhance the intervention efficacy.

### Strengths and limitations

To the best of our knowledge, this is the first meta-analysis on nonpharmacological interventions for reducing postpartum fatigue. This review was rigorous and based on the Preferred Reporting Items for Systematic Reviews and Meta-Analysis statements as well as a prospective registered protocol. Another strength was that randomized controlled trials and controlled clinical trials were included in this review, which provides good standards for evidence-based research.

There were some limitations of this meta-analysis. A limitation of the review was that non-English electronic databases were not searched, which may cause language bias [51]. The number of included studies for each type of intervention and sample sizes were small. Our results showed high heterogeneity, and the reason may be that interventions were divided into broad groups and we have pooled very different types of studies into the same category. It may have influenced the pooled results, despite using a random-effects model. Further, given the conditions of the control groups are not time-matched, hence the mechanism of change on postpartum fatigue and depression is unclear. Another limitation lies in the risk of bias within the included studies. The potential biases may have influenced the reported effect estimates; therefore, caution is required when interpreting the findings of our study.

### Future study direction

Only a part of included studies examined the effectiveness of fatigue-related interventions on depression. Given that there is a strong correlation between fatigue and depression among women in the first 2 years after child-birth [3], depression and other psychological outcomes could be included as outcomes in future research to see if fatigue-related interventions could both enhance postpartum women’s physical and psychological health. In regard to different nonpharmacological interventions for reducing postpartum fatigue, future research should work out effective and specific exercise programs for postpartum women with long term follow-up. Web-based or smartphone-based postnatal psychoeducational interventions need to be studied. Considering risk of bias within the included studies of drinking tea, risk biases existing in allocation of participants, blinding during intervention implementation and data analysis should be carefully considered. More rigorous RCTs of drinking tea should be conducted. The effectiveness of a certain physical therapy should be further investigated.

## Conclusions

The results from this meta-analysis provide evidence that nonpharmacological interventions, including exercise and drinking tea, may have immediate effect on reducing postpartum fatigue. Detailed and evidence-based instructions involving exercise frequency and duration should be offered to puerperae. RCTs with a more rigorous design examining the effect of drinking tea are needed in the future. The effects of psychoeducation were not noted, and future research could integrate psychoeducation with internet technology or smartphones. More studies should be conducted to investigate the effectiveness of a certain physical therapy. The effectiveness of fatigue-related nonpharmacological interventions on psychological outcomes still needs to be further investigated due to the limited number of studies.

## Supplementary Information

**Additional file 1.** PRISMA 2009 Checklist.

**Additional file 2.** The final search strategies.

## Data Availability

Data will be available from the corresponding author upon reasonable request.

## Abbreviations

RCT: Randomized controlled trial
CCT: Controlled clinical trial
PROSPERO: International prospective register of systematic reviews
PRISMA: Preferred reporting items for systematic reviews and meta-analyses
PICOS: Population, Intervention, Comparison, Outcome and Study design
MFI-20: Multidimensional Fatigue Inventory
PFS: Postpartum Fatigue Scale
VAS-F: visual analogue scale for fatigue
FAS: Fatigue Assessment Scale
MFSC: Modified Fatigue Symptoms Checklist
FSC: Fatigue Symptom Checklist
EDPS: Edinburgh Postnatal Depression Scale
DASS-21: Depression, Anxiety and Stress Scale-21
CES-D Scale: Chinese version of the Center for Epidemiologic Studies Depression Scale
PANAS: Positive and Negative Affect Schedule
PSS: Perceived Stress Scale
AGWBI: Adapted General Well-Being Index
CI: Confidence interval
MD: Mean difference
SMD: Standardized mean difference

## References

[1] Badr HA, Zauszniewski JA (2017). Meta-analysis of the predictive factors of postpartum fatigue. Appl Nurs Res Anr.

[2] Doering J, Durfor SL (2011). The process of “persevering toward normalcy” after childbirth. MCN Am J Matern Child Nurs.

[3] Wilson N, Lee JJ, Bei B (2018). Postpartum fatigue and depression: a systematic review and meta-analysis. J Affect Disord.

[4] Henderson J, Alderdice F, Redshaw M (2019). Factors associated with maternal postpartum fatigue: an observationalstudy. BMJ Open.

[5] Lai Y-L, Joel S, Te-Fu C, Yi L, Chich-Hsiu H (2015). Postpartum fatigue, baby-care activities, and maternal-infant attachment of vaginal and cesarean births following rooming-in. Appl Nurs Res.

[6] Rychnovsky JD (2007). Postpartum fatigue in the active-duty military woman. J Obstet Gynecol Neonatal Nurs.

[7] Kurth E, Kennedy HP, Spichiger E, Hösli I, Stutz EZ (2011). Crying babies, tired mothers: what do we know? A systematic review. Midwifery.

[8] Senol DK, Yurdakul M, Ozkan SA (2019). The effect of maternal fatigue on breastfeeding. Niger J Clin Pract.

[9] Dennis CL, Vigod S (2020). Preventing postpartum depression: fatigue management is a place to start. Evid Based Nurs.

[10] Funda Tosun G, Salime M, Özgürlük İ (2020). The effect of mother-infant skin-to-skin contact on the involution process and maternal postpartum fatigue during the early postpartum period. Women Health.

[11] Vaziri F, Shiravani M, Najib F, Pourahmad S, Salehi A, Yazdanpanahi Z (2017). Effect of lavender oil aroma in the early hours of postpartum period on maternal pains, fatigue, and mood: a randomized clinical trial. Int J Prev Med.

[12] Yang C, Chen C (2018). Effectiveness of aerobic gymnastic exercise on stress, fatigue, and sleep quality during postpartum: a pilot randomized controlled trial. Int J Nurs Stud.

[13] Ozcan S, Eryilmaz G. Using Levine's conservation model in postpartum care: a randomized controlled trial. Health Care Women In. 2020:1–21. 10.1080/07399332.2020.1797038 32744924

[14] Liu N, Wang J, Chen DD, Sun WJ, Li P, Zhang W (2020). Effects of exercise on pregnancy and postpartum fatigue: a systematic review and meta-analysis. Eur J Obstet Gynecol Reprod Biol.

[15] Doering JJ, Dogan S (2018). A postpartum sleep and fatigue intervention feasibility pilot study. Behav Sleep Med.

[16] Higgins JP, Altman DG, Gotzsche PC, Juni P, Moher D, Oxman AD (2011). The Cochrane Collaboration's tool for assessing risk of bias in randomised trials. BMJ.

[17] Sterne JA, Hernán MA, Reeves BC, Savović J, Berkman ND, Viswanathan M (2016). ROBINS-I: a tool for assessing risk of bias in non-randomised studies of interventions. BMJ.

[18] Carletta J. Assessing agreement on classification tasks: the kappa statistic. 1996;22(2):249–54.

[19] Wen J, Li Y (2007). The selection of a summary statistic for use in meta-analysis. Chin J Evid Based Med.

[20] Higgins JPT, Thompson SG (2002). Quantifying heterogeneity in a meta-analysis. Stat Med.

[21] Egger M, Smith GD, Schneider M, Minder C (1997). Bias in meta-analysis detected by a simple, graphical test. BMJ.

[22] Ashrafinia F, Mirmohammadali M, Rajabi H, Kazemnejad A, Haghighi KS, Amelvalizadeh M (2015). Effect of Pilates exercises on postpartum maternal fatigue. Singap Med J.

[23] Dritsa M, Da Costa D, Dupuis G, Lowensteyn I, Khalife S (2008). Effects of a home-based exercise intervention on fatigue in postpartum depressed women: results of a randomized controlled trial. Ann Behav Med.

[24] Ko Y, Yang C, Chiang L (2008). Effects of postpartum exercise program on fatigue and depression during “doing-the-month” period. J Nurs Res.

[25] Lee AS, McInnes RJ, Hughes AR, Guthrie W, Jepson R (2016). The effect of the more active MuMs in Stirling trial on body composition and psychological well-being among postnatal women. J Pregnancy.

[26] Gholami Z, Mohammadirizi S, Bahadoran P (2017). Study of the impact of educational behavioral interventions on fatigue in mothers in the postpartum period in the groups of face-to-face and electronic training. Iran J Nurs Midwifery Res.

[27] Giallo R, Cooklin A, Dunning M, Seymour M (2014). The efficacy of an intervention for the Management of Postpartum Fatigue. JOGNN: J Obstetr Gynecol Neonatal Nurs.

[28] Milani H, Amiri P, Mohseny M, Abadi A, Vaziri S, Vejdani M (2017). Postpartum home care and its effects on mothers' health: a clinical trial. J Res Med Sci.

[29] Stremler R, Hodnett E, Kenton L, Lee K, Weiss S, Weston J (2013). Effect of behavioural-educational intervention on sleep for primiparous women and their infants in early postpartum: multisite randomised controlled trial. BMJ.

[30] Troy NW, Dalgas-Pelish P (2003). The effectiveness of a self-care intervention for the management of postpartum fatigue. Appl Nurs Res.

[31] Chang SM, Chen CH (2016). Effects of an intervention with drinking chamomile tea on sleep quality and depression in sleep disturbed postnatal women: a randomized controlled trial. J Adv Nurs.

[32] Chen SL, Chen CH (2015). Effects of lavender tea on fatigue, depression, and maternal-infant attachment in sleep-disturbed postnatal women. Worldviews Evid-Based Nurs.

[33] Hsieh CH, Chen CL, Chung FF, Lin SY (2017). Efficacy of warm showers on postpartum fatigue among vaginal-birth Taiwanese women: a quasi-experimental design. Res Theor Nurs Pract.

[34] Larson-Meyer DE (2002). Effect of postpartum exercise on mothers and their offspring: a review of the literature. Obes Res.

[35] Adeniyi AF, Ogwumike OO, Bamikefa TR (2013). Postpartum exercise among Nigerian women: issues relating to exercise performance and self-efficacy. ISRN Obstet Gynecol.

[36] Liu N, Wang J, Chen DD, Sun WJ, Zhang W (2020). Effects of exercise on pregnancy and postpartum fatigue: a systematic review and meta-analysis. Eur J Obstet Gyn R B.

[37] Artal R, O'Toole M (2003). Guidelines of the American College of Obstetricians and Gynecologists for exercise during pregnancy and the postpartum period. Brit J Sport Med.

[38] ACOG (2015). Committee opinion no. 650 summary: physical activity and exercise during pregnancy and the postpartum period. Obstet Gynecol.

[39] Daley A, Jolly K, MacArthur C (2009). The effectiveness of exercise in the management of post-natal depression: systematic review and meta-analysis. Fam Pract.

[40] Ashish A, Sameer K, Florence D, Sara F, Jason R, Thomas U (2016). Unmet communication and information needs for patients with IBD: implications for Mobile health technology. Brit J Med Medical Res.

[41] Jiao N, Zhu L, Chong YS, Chan WS, Luo N, Wang W (2019). Web-based versus home-based postnatal psychoeducational interventions for first-time mothers: a randomised controlled trial. Int J Nurs Stud.

[42] Bevan JR, Thapar A, Rice F, Beeching H, Cichosz R, Mars B (2018). A web-based psychoeducational intervention for adolescent depression: design and development of MoodHwb. JMIR Ment Health.

[43] Chan KL, Leung WC, Tiwari A, Or KL, Ip P (2019). Using smartphone-based psychoeducation to reduce postnatal depression among first-time mothers: randomized controlled trial. JMIR Mhealth Uhealth.

[44] Yazdkhasti M, Pirak A (2016). The effect of aromatherapy with lavender essence on severity of labor pain and duration of labor in primiparous women. Complement Ther Clin Pract.

[45] Pollard K (2008). Introducing aromatherapy as a form of pain management into a delivery suite. J Assoc Chart Physiother Womens Health.

[46] Cavanagh HMA, Wilkinson JM (2002). Biological activities of lavender essential oil. Phytother Res.

[47] Stark MA (2013). Therapeutic showering in labor. Clin Nurs Res.

[48] Fatemeh ED, Sakineh MAC, Mojgan M, Mohsen T, Reza B, Somayeh Z (2018). Effect of lavender cream with or without footbath on sleep quality and fatigue in pregnancy and postpartum: a randomized controlled trial. Women Health.

[49] Kirca N, Adibelli D. Effects of mother–infant skin-to-skin contact on postpartum depression: a systematic review. Perspect Psychiatr C. 2021:1–10. 10.1111/ppc.12727. 10.1111/ppc.12727 33476428

[50] Son CM, Ja LE (2015). Effects of foot-reflexology massage on fatigue, stress and postpartum depression in postpartum women. J Korean Acad Nurs.

[51] Morrison A, Polisena J, Husereau D, Moulton K, Clark M, Fiander M (2012). The effect of English-language restriction on systematic review-based meta-analyses: a systematic review of empirical studies. Int J Technol Assess.

