# Supplementary material for: Effectiveness of nonpharmacological interventions for reducing postpartum fatigue: a meta-analysis
Source: BMC Pregnancy Childbirth. 2021 Sep 14;21:622. doi: 10.1186/s12884-021-04096-7 (PMC8442348; doi:10.1186/s12884-021-04096-7)
Supplement: Supplementary file 2 — Additional file 2. The final search strategies. [file 12884_2021_4096_MOESM2_ESM.doc]

**Additional file 2 The final search strategies**

| Database | Search strategies | Results |
| --- | --- | --- |
| Cochrane Library | 1. postpartum period[MeSH Terms] 2. postpartum period 3. postpartum 4. postnatal 5. delivery 6. childbirth 7. birth 8. parturition 9. labour 10. labor 11. pregnancy 12. OR/1-11 13. fatigue[MeSH Terms] 14. fatigue 15. mental fatigue[MeSH Terms] 16. mental fatigue 17. lassitude 18. exhaust* 19. tiredness 20. tired* 21. OR/13-20 22. randomized controlled trial 23. controlled clinical trial 24. cohort 25. clinical trial 26. OR/21-24 27. 12 AND 21 AND 26   Filters: Trials | 1091 |
| PubMed | 1. postpartum period[MeSH Terms] 2. postpartum period[Title/Abstract] 3. postpartum[Title/Abstract] 4. postnatal[Title/Abstract] 5. delivery[Title/Abstract] 6. childbirth[Title/Abstract] 7. birth[Title/Abstract] 8. parturition[Title/Abstract] 9. labour[Title/Abstract] 10. labor[Title/Abstract] 11. pregnancy[Title/Abstract] 12. OR/1-11 13. fatigue[MeSH Terms] 14. fatigue[Title/Abstract] 15. mental fatigue[MeSH Terms] 16. mental fatigue[Title/Abstract] 17. lassitude[Title/Abstract] 18. exhaust*[Title/Abstract] 19. tiredness[Title/Abstract] 20. tired*[Title/Abstract] 21. OR/13-20 22. randomized controlled trial[Publication Type] 23. controlled clinical trial[Publication Type] 24. cohort [Title/Abstract] 25. clinical trial[Publication Type] 26. OR/22-25 27. 12 AND 21 AND 26   Filters: Clinical Trial, Randomized Controlled Trial | 357 |
| Embase | 1. 'puerperium'/exp 2. 'postnatal':ab, ti 3. 'postpartum'/exp 4. 'obstetric delivery'/exp 5. 'childbirth'/exp 6. 'birth'/exp 7. 'labor'/exp 8. 'pregnancy'/exp 9. OR/1-8 10. 'fatigue'/exp 11. 'mental fatigue':ab, ti 12. 'lassitude'/exp 13. 'exhaust*':ab, ti 14. 'tiredness':ab, ti 15. 'tired*':ab, ti 16. OR/10-15 17. 'randomized controlled trial'/exp 18. 'controlled clinical trial'/exp 19. 'cohort':ab,ti 20. 'clinical trial'/exp 21. OR/15-18 22. 9 AND 16 AND 21 23. Publication types: Article | 416 |
| Web of Science | 1. postnatal OR postpartum OR delivery OR childbirth OR birth OR parturition OR labour OR labor OR pregnancy 2. fatigue OR mental fatigue OR lassitude OR exhaust* OR tiredness OR tired* 3. randomized controlled trial OR controlled clinical trial OR cohort OR clinical trial 4. 1 AND 2 AND 3 5. Publication Type: CLINICAL TRIAL | 345 |
| PsycINFO | 1. (postnatal OR postpartum OR delivery OR childbirth OR birth OR parturition OR labour OR labor OR pregnancy)[Abstact] 2. (fatigue OR mental fatigue OR lassitude OR exhaust* OR tiredness OR tired*)[Abstact] 3. (randomized controlled trial OR controlled clinical trial OR cohort OR clinical trial)[Abstact] 4. 1 AND 2 AND 3 | 114 |
| CINAHL | 1. (postnatal OR postpartum OR delivery OR childbirth OR birth OR parturition OR labour OR labor OR pregnancy)[Abstact] 2. (fatigue OR mental fatigue OR lassitude OR exhaust* OR tiredness OR tired*)[Abstact] 3. (randomized controlled trial OR controlled clinical trial OR cohort OR clinical trial)[Abstact] 4. 1 AND 2 AND 3 | 238 |
| ProQuest | 1. (postnatal OR postpartum OR delivery OR childbirth OR birth OR parturition OR labour OR labor OR pregnancy)[Abstact] 2. (fatigue OR mental fatigue OR lassitude OR exhaust* OR tiredness OR tired*)[Abstact] 3. (randomized controlled trial OR controlled clinical trial OR cohort OR clinical trial)[Abstact] 4. 1 AND 2 AND 3 | 420 |
| OpenGrey | Postpartum fatigue | 0 |
